# A scoping review of Do-It-Yourself Automated Insulin Delivery system (DIY AID) use in people with type 1 diabetes

**DOI:** 10.1371/journal.pone.0271096

**Published:** 2022-08-11

**Authors:** Amy E. Morrison, Kimberley Chong, Peter A. Senior, Anna Lam

**Affiliations:** 1 Division of Endocrinology and Metabolism, Department of Medicine, University of Alberta, Edmonton, AB, Canada; 2 Alberta Diabetes Institute, University of Alberta, Edmonton, AB, Canada; University of Milano Bicocca, ITALY

## Abstract

**Introduction:**

User designed Automated Insulin Delivery systems (AID), termed Do-It-Yourself (DIY) AID include; AndroidAPS, OpenAPS and Loop. These unregulated systems provide challenges for healthcare providers worldwide, with potential legal and ethical barriers to supporting their use. We performed a scoping review of the currently available literature surrounding DIY AID systems, specifically to highlight the evidence available to facilitate healthcare providers to support persons with diabetes who may benefit from DIY AID.

**Methods:**

Studies relating to DIY AID systems were searched in Embase, Medline, Web of Science, Scopus, Proquest and Cochrane library until 31^st^ December 2021. Publications were screened through title and abstract to identify study type and AID system type described. A thematic synthesis methodology was used for analysis of studies of DIY AID use due to the heterogeneity in study designs (case reports, qualitative, cross-sectional and cohort studies), with similarity in outcome themes.

**Results:**

Following implementation of the search strategy, 38 relevant full texts were identified; comprising 12 case reports, 9 qualitative studies and 17 cohort studies, and data was also available from 24 relevant conference abstracts. No randomized studies were identified. Common themes were identified in the outcomes across the studies; glycemic variability, safety, quality of life, healthcare provider attitudes and social media.

**Conclusion:**

There is extensive real-world data, but a lack of randomized control trial evidence supporting DIY AID system use, due to the user-driven, unregulated nature of these systems. Healthcare providers report a lack of understanding surrounding, and confidence in supporting, DIY AID despite impressive observational and user self-reported improvements in glycemic variability, without any reported safety compromises.

## Introduction

Do-It-Yourself (DIY) or Open-Source Automated Insulin Delivery (AID) systems combine a Continuous Subcutaneous Infusion of Insulin (CSII) via an insulin pump, with a Continuous Glucose Monitor (CGM), through a user-built computerized algorithm to enable automated adjustment in insulin delivery rate. These closed loop systems, which do not have regulatory approval, are categorized by the technology and algorithm they incorporate. AndroidAPS, OpenAPS and Loop are the prominent system sub-types [1], with more recent utilization of similar algorithms combined with different devices such as the FreeAPSX branch [2]. These systems are rapidly gaining in popularity worldwide with a notable social media presence through the #WeAreNotWaiting movement [3]. People with type 1 diabetes are choosing to use these systems to enable flexible self-management of their condition, with a desire for improved quality of life [4].

Improved glycemic and long-term health outcomes are the two most frequently reported motivating factors for individuals choosing to use a DIY system [5]. The traditional measure of glycemia; Glycated hemoglobin (HbA1c), an assessment of the preceding three months glucose levels, provides a marker of risk for the development of long-term diabetes-related complications [6]. More recently, the introduction of Continuous Glucose Monitoring (CGM) has enabled assessment of day-to-day glycemia in an easy to interpret format for the user. This has led to the introduction of three key concepts; time in range (TIR), time above range (TAR) and time below range (TBR), to further assess glycemic variability. TIR refers to the proportion of time that a person spends with their glucose levels within a specified target range, usually 3.9–10 mmol/L. Consensus recommendations suggest aiming for TIR >70%, corresponding with an HbA1c of 7%. Suggested targets for TAR and TBR are <25% and <4%, with targets for both proportion of time and glucose levels needing adjustment dependent upon individual factors, notably in the elderly, higher risk people with diabetes, and during pregnancy [7]. DIY AID users suggest these systems support them to reach these recommended glycemic targets and to therefore minimize development of diabetes-related complications [4, 6].

Despite these striking benefits for users, Healthcare providers (HCP) worldwide are challenged by the novel and unregulated approach to diabetes care that DIY AID poses. These are systems which the majority of HCP have limited experience with, and unlike most healthcare processes, have been instigated and set up by the person with diabetes, rather than their healthcare team [3]. Providing the technology and medical supplies required for the continued use of these systems, with the knowledge that their patients are using them in an unregulated way, has potential ethical implications for HCP [8]. The uncertainty surrounding ethical, legal and liability considerations continue to result in inconsistent care for people with diabetes choosing to use these systems worldwide [9].

Conflicting guidance for professionals regarding DIY AID use has been issued by specialist diabetes networks in Europe and Australia [10–13], with some recommending prioritization of patient choice and support [10, 12], while others highlight the prospects of criminal and liability issues if actively supporting patient’s use of these systems [13]. The need for further outcome studies in the use of these systems has been highlighted as a priority by Diabetes Poland, with safety as a primary outcome, in order to enable physicians to support their patients to achieve the associated benefits of DIY AID, with the suggestion of hospitals or camps as potential safe locations to perform these novel studies [12]. More recently, in an attempt to rectify this uncertainty, the OPEN International Healthcare Professional Network and OPEN Legal advisory group have published a consensus statement, with practical guidance for HCP in the use of DIY or open-source AID [14]. This group, comprising specialist HCP and legal experts in the field of AID systems make reference to the challenges in supporting users of an unregulated system and the need to take into account local law and organizational governance in clinical practice.

We performed a scoping review of the currently available literature surrounding DIY AID systems, specifically to highlight the evidence available to surrounding their use. We aimed to identify studies reporting on the impact of DIY AID systems on type 1 diabetes management for both users and their care givers, or HCP, with the goal of collating outcome data; glycemic variability, safety and quality of life. In addition, we hoped to gain a greater understanding of the experiences of DIY AID system users and HCP providing care for people using these systems.

## Methods

This review was carried out in accordance with the Enhancing Transparency in Reporting the Synthesis of Qualitative Research (ENTREQ) criteria [15] (Table 1.4, in S1 Appendix). A thematic synthesis methodology was used for analysis; with heterogeneity seen in the incorporated study designs (case reports, qualitative, cross-sectional and cohort studies), but consistency in the reported outcome measures. This methodology enables translation of concepts across studies, with organization of descriptive themes and transparency in analysis of the study results [16].

### Data sources and searches

**Fig 1:**
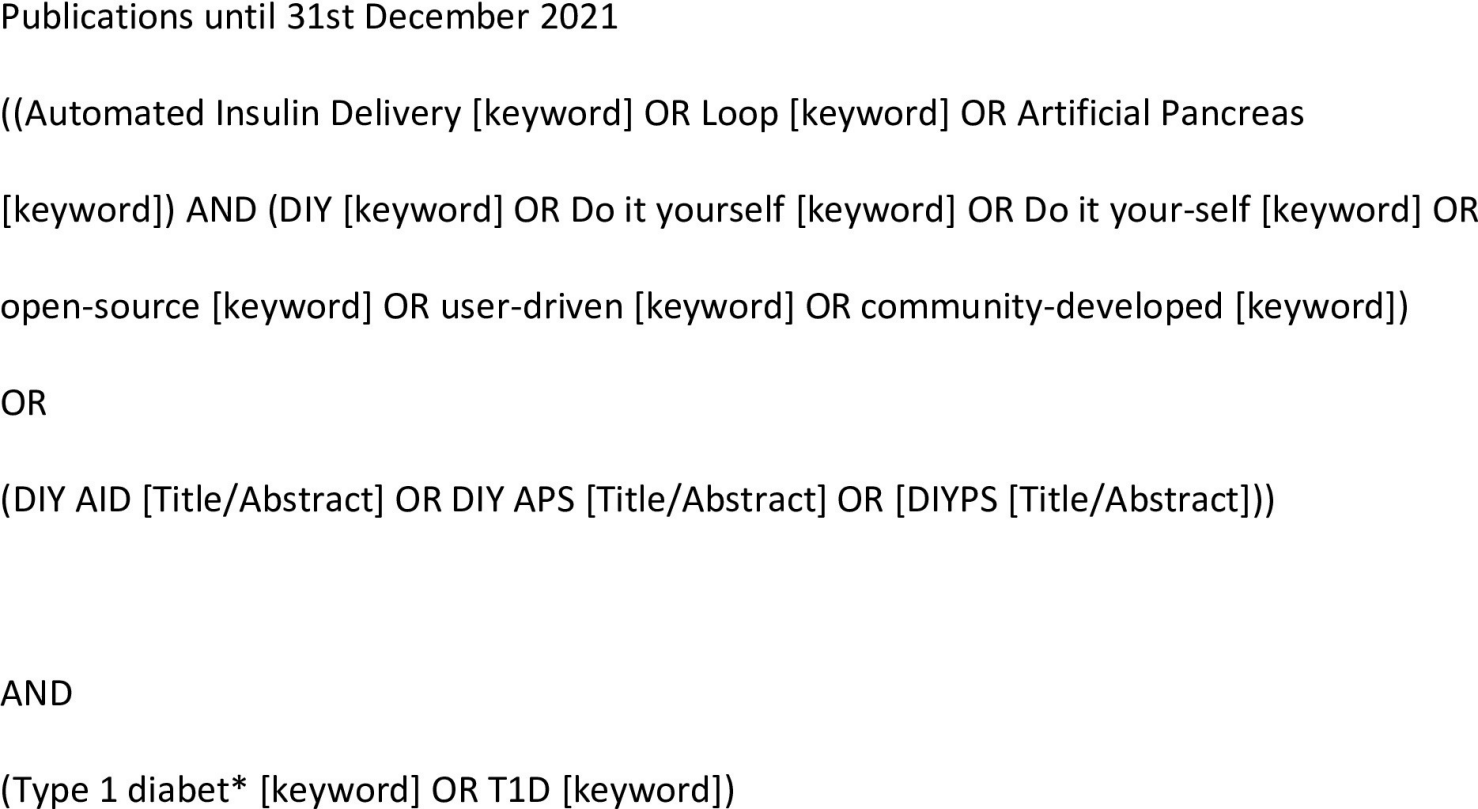
Search strategy.

A review was conducted of the available literature in the use of DIY AID systems, published until 31^st^ December 2021. Relevant articles published in English were systematically sought using the databases Embase, Medline, Web of Science, Scopus, Proquest and Cochrane library. Terms were searched as keywords within the title or abstract of the manuscript, combining the description of the disease of interest (type 1 diabetes) and those used in the description of DIY AID systems (do-it-yourself, loop, automated insulin delivery or artificial pancreas system), the full search strategy is shown in Fig 1. Additional grey literature searches took place through the search engine ‘Google’, reviewing the first 10 pages of results in the search ‘DIY’ and ‘type 1 diabetes’ on 31^st^ December 2021. The chronological summary of DIY AID system outcome data available at openAPSoutcomes.org was additionally reviewed [4]. Conference abstracts were identified, in addition to those highlighted in the above searches, through review of the available listings for the past two years at the American Diabetes Association (ADA 2019, 2020), Diabetes UK (DUK 2020, 2021) and Advanced Technologies & Treatments for Diabetes (ATTD 2020, 2021) conferences, specifically to identify additional relevant data, not yet formally published.

### Study selection

**Fig 2:**
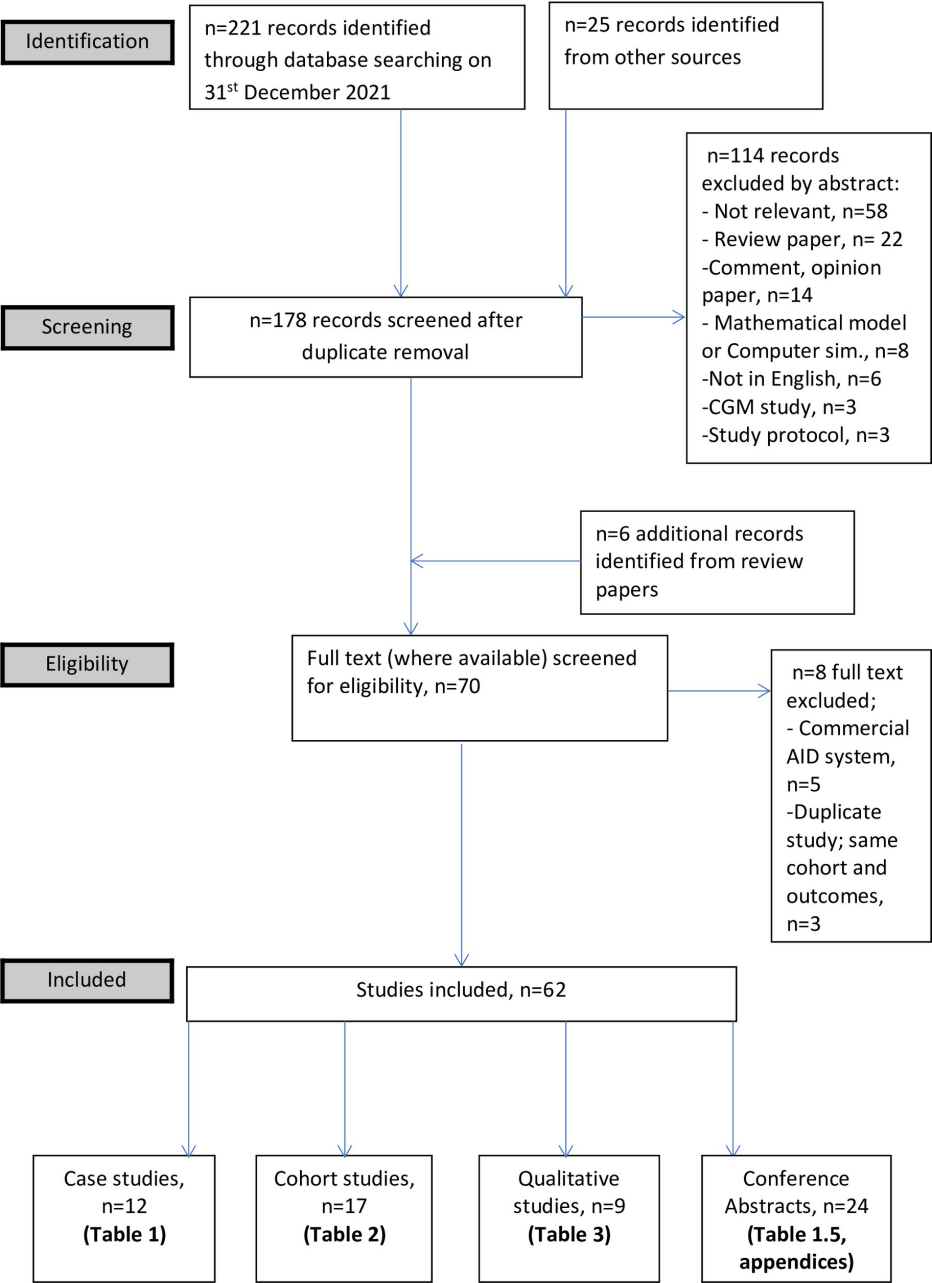
PRISMA flowchart.

Two independent reviewers (AM and KC) screened the identified titles after implementing the search strategy (Fig 1), for those deemed to be relevant, review of abstract and full text (where available) took place. Article type and which AID system used were assessed. Case reports/case series, qualitative studies, prospective cohorts, retrospective cohorts, cross-sectional studies and conference abstracts were included for review (PRISMA Flowchart in Fig 2). Only studies in humans, published in English, reporting on the use of the DIY AID systems; OpenAPS, AndroidAPS or Loop were included in the results. Reference lists of both included studies and review articles were screened for any additional relevant studies requiring inclusion into this review.

### Quality assessment

The identified cohort group DIY AID studies were sub-classified according to the data collection method; into qualitative, prospective and retrospective cohort or cross-sectional studies. Quality assessment of full text, excluding case report studies, was carried out using the Clinical Appraisal Skills Program checklist for qualitative and cohort studies (Tables 1.1 and 1.2 in S1 Appendix) and AXIS (the Appraisal Tool for Cross-Sectional studies) for the identified cross-sectional studies (Table 1.3 in S1 Appendix). These are validated checklists, according to study type, looking broadly at the domains of aims, methodology, results, analysis, overt findings and implications of these on future practice, with each domain assessed as ‘yes’, ‘no’ or ‘can’t tell’ [17–19].

### Analysis

For all relevant study and text types identified, reporting qualitative or quantitative results in the use of DIY AID systems, data was extracted on; first author, year of publication, geographical area, study methodology, participants, outcomes and measurement of these outcomes. Common themes were identified in the outcomes reported across the DIY AID cohort studies and the results reported within these outcome themes. The themes identified comprised; glycemic variability, safety, quality of life, healthcare provider attitudes and social media. Glycemic data are reported using a standardized TIR of 3.9–10.0 mmol/L, unless otherwise stated.

## Results

Following implementation of the search strategy (Fig 1), 244 potentially relevant articles were reduced down to 70 full texts, after exclusion through screening of title and abstract (PRISMA, Fig 2). Following detailed assessment, 62 studies reporting on the use of DIY AID systems were included in our results, these comprised; 12 case reports/case series, 17 cohort studies, 9 qualitative studies and 24 conference abstracts.

Quality assessment revealed high-quality study design across the included study types with quality consideration throughout study aims, methodology, recruitment processes, consideration of ethical issues, data analysis and reporting of findings. However, in 8 of the 9 reported qualitative studies it was unclear as to whether the relationship between the researcher and participant had been considered by the study team. Of the included cohort studies, 6 studies identified possible confounders in outcomes, but none of these considered confounders in data analysis. The key deficiency highlighted in cross-sectional study quality was a lack of consideration of response bias, identified in 3 of the 8 studies.

### DIY AID case reports

The main characteristics of the case report studies are shown in Table 1 [20–31].

**Table 1:** Case control studies [20–31].

**First Author (Year)**	**Country (PMID)**	**System**	**Participants**	**Outcome Measure and Results**	
Marshall (2019)	UK (31440989)	1. AndroidAPS 2. OpenAPS 3. Loop	3 patient experiences; 2 male, 1 female during pregnancy	HbA1c (mmol/mol), TIR with DIY AID (using TIR 4–10 in 1, 3.6–14 in 2 and 3.5–7.8 mmol/mol in 3)	1. 43, 85–90% 2. 45.4, 91% 3. 42, 80%
Patton (2019)	Australia (n/a)	OpenAPS	50 yr. female, with 38-year history of T1D	Glycemic outcomes Qualitative impact on day-to-day life	HbA1c reduction to 6%, increased TIR. QoL benefits; usability of technology and convenience.
Duke (2020)	USA (32131623)	Loop	Parent perspective starting Loop for son	HbA1c Loop vs pre-DIY Quality of Life	6.3 vs 8.1% Family feel ‘more rested and more balanced and are able to think about something other than diabetes’
Braune (2020)	Germany (31709805)	AndroidAPS for 23 months	49-year-old male, T1D for 32 years, running a half marathon	Race completion TIR during race Hypoglycaemia	Race completed in 1hr 52 mins 100% No hypoglycemia
Schutz-Fuhrmann (2020)	Austria (32059616)	Pregnancy 1- MDI and Flash glucose monitoring Pregnancy 2-AndroidAPS	37-year-old female, during two pregnancies; Pregnancy 1 age 35 years Pregnancy 2 age 37 years	Pregnancy 2 with AndroidAPS; HbA1c Trimester (TM) 1 TM2 TM3 TIR (63-140mg/dL) TM 1 TM2 TM3 TBR TM1 TM2 TM3 Birth weight	vs Pregnancy 1 with MDI and Flash GM 6.3 vs 5.9% 5.1 vs 5.1% 5.0 vs 4.9% 74 vs 51% 76 vs 71% 77 vs 69% 9 vs 12% 12 vs 13% 14 vs 13% 2900 vs 2820g
Ahmed (2020)	UK (32696329)	1. Loop 2. Loop 3. AndroidAPS	1. 31-year-old female 2. 18-year-old male 3. 10-year-old male	HbA1c, TIR with DIY AID	vs previous insulin delivery 1. 5.5 vs 6.5%, 90–95% 2. 42 vs 70mmol/mol, 80% vs 60% 3. Glycemic data not available
**First Author (Year)**	**Country (PMID)**	**System**	**Participants**	**Outcome Measure and Results**	
Ahmed (2020)	UK (32922559)	Loop	Muslim female with T1DM fasting during Ramadan	CGM data during the month of Ramadan. Comparative experience to Medtronic 670G use previously	Enabled this person with diabetes to fast during Ramadan with customizable settings
Lemieux (2021)	Canada (33648862)	1. Loop 2. OpenAPS	1.31-year-old female Loop use from 13 months pre-pregnancy 2.40-year-old female- OpenAPS use from 3 months pre-pregnancy	HbA1c pre-conception, TIR (3.5–7.8mmol/L) TM 1/2/3, TBR TM1/2/3, Mean HbA1c during pregnancy, Delivery; Date, Birth Weight user 1 and 2.	1. 6.2%, 71.6/72.9/81.3%, 4.6/4.1/2.3%, 5.7%, 38+5, 3070g 2. 5.1%, 87.5/86.6/89.1%, 1.9/1.8/2.0%, 5.0% 39+1, 3440g
Kesavadev (2021)	India (33725629)	Loop	24-year-old female	With Loop use; HbA1c TIR Fear of hypoglycemia and QOL	vs CSII 6.2 vs 7.2% 90–95 vs <60% Improvements in HFS-II and Dqol with Loop
Waiker (2021)	USA (34866788)	Loop	30-year-old female, Loop use pre conception and during pregnancy	One month pre- conception; HbA1c TIR (70-180mg/dL) TBR TM 1/2/3 TIR (64-140mg/dL) TBR Time <54mg/dL Pregnancy outcomes including birth weight	6.0% 77% 5% 62.8/66/76.5% 3.7/4.6/4.3% 0.9/2.1/1.1% Induction 39/40 with unplanned C-section, BW = 2910g, 24hrs in NICU–hypoglycemia
**First Author (Year)**	**Country (PMID)**	**System**	**Participants**	**Outcome Measure and Results**	
Schutz (2021)	Austria (34542371)	Loop (3 users) AndroidAPS (1 user)	3 Loop users during pregnancy; 36, 26, 28 years old. 1 AndroidAPS user during pregnancy; 39 years old.	Pre-pregnancy HbA1c (user 1,2,3,4) TIR (63-140mg/dL) TM1 TM 2 TM 3 Pregnancy outcomes; date at delivery (weeks), birth weight.	5.7, 5.9, 6.2, 6.5% 78.4, 77, 61.3, 74% 85.8, 80.4, 78.6, 76.0% 88.8, 82.2, 82.0, 77% 40, 37+4, 39+1, 38+5 3040, 3750, 3600, 2900g
Bukhari (2021)	USA (34535491)	Loop	40-year-old female, Loop use during pregnancy and 6 months pre-conception	Pre-conception HbA1c TIR TM 1/2/3 TBR TAR Birth weight Type and timing of delivery	6.4% 66/68/72% 6/5/7% 28/27/21% 3742g Emergency C-section, 37 weeks, no neonatal complications

These 12 articles, report DIY AID use in a total of 20 individuals (70% female); 12 Loop, 5 AndroidAPS and 3 OpenAPS. The study outcomes report HbA1c (% or mmol/mol) in 10 studies, with HbA1c value pre-DIY system use additionally available in 5 of these studies, with 80% reporting an improvement in HbA1c with DIY AID use. TIR is a reported outcome in 10 of the studies, with pre-DIY AID TIR values available in 2, and an improvement in TIR with DIY AID use demonstrated in both of these. Diabetes related quality of life score (dQOL) and fear of hypoglycaemia (HFS-II) were reported outcomes in one case report, with improvements seen with Loop use [28].

Pregnancy use of DIY AID is reported in 10 individuals with; Loop (n = 7), AndroidAPS (n = 2) and OpenAPS (n = 1) [24, 27, 29–31]. TIR according to pregnancy targets (3.5–7.8mmol or 63-140mg/dL) was > 70% in every trimester in seven of these nine individuals. One study compared glycemic outcomes for the same individual in a pregnancy using AndroidAPS to a previous pregnancy managed using multiple daily injections (MDI) of insulin, with improvement in TIR with the use of the DIY AID system in each trimester. TIR vs previous pregnancy; trimester 1–74 vs 51%, trimester 2–76 vs 71% and trimester 3–77 vs 69%. TBR (<63mg/dL) was superior in the first trimester and similar for the rest of pregnancy, TBR vs previous pregnancy; trimester 1–9 vs 12%, trimester 2–12 vs 13% and trimester 3–14 vs 13% [24].

Case reports additionally discussed the beneficial impact of DIY AID use in people with type 1 diabetes in situations where maintaining stable blood glucose levels would be challenging; running a half marathon [23] and fasting during Ramadan [26].

### Cohort studies

The 17 cohort studies relating to DIY AID use, described in Table 2 [5, 32–47], comprised cross-sectional (n = 8), retrospective (n = 6) and prospective (n = 3) studies, relating to the use of AndroidAPS (n = 4), OpenAPS (n = 2), Loop (n = 1) or a combination of the three system types (n = 10).

**Table 2:** DIY AID cohort studies [5, 32–47].

**First Author (Year)**	**Country (PMID)**	**AID System (Study type)**	**Participants**	**Outcome Measure and Results**	
Lewis (2016)	USA (27510442)	OpenAPS (Retrospective cohort)	18 users; 12 male, 6 female. Median; age 27yrs, 15 years of diabetes, 10 years Continuous Subcutaneous Insulin Infusion (CSII) and 3 years CGM use.	Self-report measures: HbA1c TIR (80-180mg/dL) Improved sleep quality	vs pre OpenAPS 6.2 vs 7.1% 81 vs 58% 94% reported
Hng (2018)	Australia (30387315)	OpenAPS, AndroidAPS and Loop (Cross- sectional)	Online survey posted to Australian Loop Facebook group. 68 respondents, 20 Loopers, 4 carers of Loopers.	Loopers (%) reported improvements in; TIR Sleep Hypoglycaemia frequency HbA1c, Confidence Energy Mood swings	100 79 74 68 47 37 32
Petruzelkova (2018)	Czech Republic (30285476)	AndroidAPS vs SAP (Prospective cohort)	22 children, 6–15 years, 16 female, 6 male during alpine ski sports camp, for three days and nights.	With AndroidAPS; Mean glucose level TBR TIR	vs SAP *Clinician collected data 7.2 vs 7.7 mmol/L (p = 0.042) 5 vs 3% (p = 0.6) 82 vs 82% (p = 0.3)
Melmer (2019)	Switzerland (31183929)	OpenAPS (Retrospective cohort)	Analysis of anonymized CGM records of 80 users uploaded to the OpenAPS Data Commons repository; 19495 days or 53.4 years of total data. 34 of the users had additional CGM data when previously using Sensor Augmented Pump (SAP) to compare.	With OpenAPS; Mean glucose TIR TBR <3.0mmol/L >10mmol/L >13.9 mmol/L Change relative to SAP; mean glucose, HbA1c TIR <3.0mmol/L Coefficient of variation	7.6mmol/L 77.5% 4.3% 1.3% 18.2% 4.1% -0.6mmol/L (p<0.0001) -0.4% (p<0.0001) +9.3% (p<0.0001) -0.7% (p = 0.0171) -2.4% (p = 0.0198)
**First Author (Year)**	**Country (PMID)**	**AID System (Study type)**	**Participants**	**Outcome Measure and Results**	
Braune (2019)	Germany with virtual survey respondents from 21 countries (31364599)	AndroidAPS (48%) OpenAPS (28.4%) Loop (28.4%) (Retrospective cohort)	Online survey distributed via Facebook groups; 209 participants, 47.4% female, median age 10 years (range 3–20), median duration DIYAID 7.5months. Self-reported outcomes by person with diabetes or caregivers pre and post DIYAID use.	Mean HbA1c after initiation; ALL DIY AndroidAPS OpenAPS Loop Mean TIR after initiation; ALL DIY AndroidAPS OpenAPS Loop	vs pre-DIY 6.27 vs 6.91% (p<0.001) 6.24 vs 6.85% 6.36 vs 7.1% 6.39 vs 6.99% 80.68 vs 64.2% (p<0.001) 79.5 vs 63.8% 81.7 vs 67.1% 79.1 vs 64.2%
Murray (2020)	USA (31876176)	AndroidAPS, OpenAPS and Loop (Cross-sectional)	Phase 1 –paper-based survey, 43 HCPs, 90.7% female. Phase 2- online survey, 137 HCPs, 93% female, 91% nurses and nutritionists.	HCP experiences with DIY and Commercial AID, barriers to answering questions about DIY AID.	11.6% (DIY), 34.9% (Commercial) comfortable answering questions relating to these systems, 74.4% report lack of understanding how DIY AID systems work.
Crabtree (2020)	UK (32085825)	AndroidAPS, OpenAPS and Loop (Cross-sectional)	Survey Monkey link for HCP, 317 respondents; 46% consultants, 38% diabetes specialist nurses or dieticians, 27% HCPs in paediatrics.	Initiation of conversations about DIYAPS and reasons why, perception of DIYAPS as dangerous, willingness to support users and learn more about DIYAPS.	91% would not initiate conversations, 2% perceived DIYAPS as dangerous, 55% willing to support users, 97% wished to learn more about DIYAPS.
Palmer (2020)	USA (32680447)	AndroidAPS, OpenAPS and Loop (Cross-sectional)	User survey via Facebook and Twitter; 101 participants. HCP survey via the American Association of Diabetes Educators; 152 participants.	User self-reported glycemic control and safety. HCP perception safety DIY AID.	94% patients reported improved TIR. 89% users reported the systems to be safe, relative to 27% HCP.
**First Author (Year)**	**Country (PMID)**	**AID System (Study type)**	**Participants**	**Outcome Measure and Results**	
Herzog (2020)	Germany (33332410)	AndroidAPS, OpenAPS and Loop (Cross-sectional)	Survey of 1054 people with diabetes, 86 respondents using DIY closed loop; 92% using AndroidAPS.	Mean self-reported TIR DIY Reported HbA1c improvement using DIY AID. Positive perceived aspects of DIY AID Negative aspects DIY	79.5% 91% stated HbA1c improvement 43.8% better TIR, 22.5% better sleep quality, 17.9% fewer hypoglycemic episodes, 10.1% better disease management. Complexity of system 14.6%, lack of institutional approval 4.5%.
Wu (2020)	China (32922721)	AndroidAPS (Retrospective cohort)	15 participants; 10 females, median age 32.2 years, diabetes duration 9.7years with a minimum of 3 months continuous AndroidAPS use after SAP at baseline.	After 3 months AndroidAPS; HbA1c Mean glucose TIR TBR Fear of hypoglycaemia, Diabetes distress (little/no distress), EQ-5D-5L VAS	vs SAP at baseline 6.79 vs 7.63% (p = 0.02) 7.43 vs 8.03 mmol/L (p<0.001) 84.28 vs 75.01% (p<0.001) 1.72 vs 2.83% (p = 0.011) 22.13 vs 26.27 points, max 72 (p = 0.01) 9 vs 6% (p = 0.143) 82 vs 77 points, max 100 (p = 0.130)
Lum (2021)	USA (33226840)	Loop (Prospective cohort)	558 new Loop users (<7days), age range 1–71 years, observational study with 6 months CGM data.	With 6 months Loop use; TIR Mean glucose HbA1c TBR	vs baseline 73 vs 67% (p<0.001) 147 vs 155 mg/dL (p<0.001) 6.5 vs 6.8% (p<0.001) 2.8 vs 2.9% (p = 0.002)
Petruzelkova (2021)	Czech Republic (33576551)	AndroidAPS (Retrospective cohort)	36 children; 18 pre-school (age 3–7 years), 18 school age (age 8–14) who had switched from SAP to AndroidAPS.	After 6 months AndroidAPS; HbA1c TIR 3–3.8mmol/L	pre-school vs SAP and school age children vs SAP 48.5 vs 53.8mmol/mol (p = 0.001) and 45.1 vs 52.6mmol/mol (p = 0.001) 78.6 vs 70.8% (p = 0.004) and 82.9 vs 77.2% (p<0.001) 3.0 vs 3.0% (p = 0.9) and 3.8 vs 2.6% (p = 0.04)
**First Author (Year)**	**Country (PMID)**	**AID System (Study type)**	**Participants**	**Outcome Measure and Results**	
Gawrecki (2021)	Poland (33819289)	AndroidAPS (Prospective cohort)	12 subjects; 5 men, 7 women, mean age 31.3 years, duration of diabetes 16.1 years, HbA1c 6.8%/51.3 mmol/mol on CSII at baseline, after 3-week run-in period, 12 weeks of AndroidAPS use.	After 12 weeks AndroidAPS; TBR TIR <70mg/dL HbA1c Insulin requirement Body weight Safety	vs baseline *Clinician collected data 0.35 vs 0.25% (not significant) 79.3 vs 68% (p<0.001) 1.75 vs 2.50% (ns) 6.3 vs 6.8% (p<0.001) 0.60 vs 0.62 units/kg (ns) 71.3 vs 70.5kg (ns) No Severe hypoglycemia /DKA with AndroidAPS
Jeyaventhan (2021)	UK (33999488)	Loop, AndroidAPS, OpenAPS vs Medtronic 670G (Retrospective cohort)	68 participants; 38 Medtronic 670G, 30 DIY (50% Loop, 36.7% AndroidAPS, 13.3% OpenAPS). 6 months of glycemic data reviewed with respective systems.	Change with 6 months DIY; HbA1c TIR Mean glucose TAR TBR Safety	vs 6 months Medtronic 670G use *Clinician collected data -0.9 vs -0.1% (p = 0.04) 78.5 vs 68.2% (p = 0.024) 7.6 vs 8.9 mmol/L (p = 0.024) 18.4 vs 29.2% (p = 0.24) 3.2 vs 2.6% (p = 1) A non-significant increase severe hypoglycemia with 670G, (p = 0.104) No DKA in either group
March (2021)	USA (33900843)	OpenAPS AndroidAPS Loop (Cross-sectional)	104 school nurses, completed online survey of current practices, knowledge and beliefs surrounding DIY AID; 99% female, mean age 47.9 years.	Have a student using DIY AID No prior knowledge of DIY AID Children should be able to use DIY AID in school. School nurse should be responsible for DIY system if child not independent. Students should be able to share CGM data with parent/guardian. Open-ended question response themes.	23% 46% 82% 33% 96% Guidance and defined expectations, reactions to fears and the unknown, adopt and adapt.
**First Author (Year)**	**Country (PMID)**	**AID System (Study type)**	**Participants**	**Outcome Measure and Results**	
Braune (2021)	Germany with virtual respondents from 35 countries (34096874)	OpenAPS AndroidAPS Loop (Cross-sectional)	897 participants; 722 adults with T1DM, 175 caregivers of children with T1DM. Web-based cross-sectional survey (DIWHY)	Motivations to commence OpenAPS for Adult users Self-reported; HbA1c TIR	vs caregivers Improve glycemic control 93.5% vs 95% Reduce acute complications 97.2% vs 96% Reduce LT complications 83.3% vs 91% Less freq. tech interaction 81.1% vs 86% Improved sleep quality 71.6% vs 80% Increased life expectancy 75.1% vs 84% Lack of Commercial AID 70.8% vs 80% Not reaching goal with available therapy 68.4 vs 69% (vs pre-DIY AID) 6.24 vs 7.14% 80.34 vs 62.96%
Street (2021)	UK with virtual respondents (34047963)	AndroidAPS (65.6%), Loop (30.4%) and OpenAPS (3.2%) (Cross-sectional)	296 participants (253 from UK) in an online survey distributed via Twitter and Facebook groups (Looped and AndroidAPS users); 43.1% female, median age 35 years, duration diabetes 19.5 years, average duration DIY AID 10.3 months.	User demographics, type and duration of DIY AID use. TIR change with DIY AID use.	Peak ages 10–15 years and 40–45 years. Average age; Loop user 28.5 years AndroidAPS 35.8 years OpenAPS 33 years Mean increase TIR 17.3%.

HCP opinions on DIY AID systems were reported in 4 studies, and 15 studies reported user opinions and/or their outcomes with DIY AID use. The USA contributed to the production of the greatest number of cohort studies (n = 5, 29%), in addition to Germany (n = 3), United Kingdom (n = 3), Czech Republic (n = 2), Switzerland (n = 1), Australia (n = 1), China (n = 1) and Poland (n = 1).

### Qualitative studies

The 9 qualitative studies identified relating to DIY AID use, described in Table 3 [48–56], comprised interview studies (n = 5), analysis of Twitter data (n = 2), a workshop summary (n = 1) and analysis of study coordinator meetings (n = 1).

**Table 3:** DIY AID qualitative studies [48–56].

**First Author (Year)**	**Country (PMID)**	**AID System (Study type)**	**Participants**	**Outcome Measure and Results**	
Litchman (2019)	USA (30198751)	OpenAPS (Qualitative)	3347 tweets by 328 OpenAPS users, care givers and care partners	Twitter perceptions of OpenAPS use	1. Self-reported HbA1c and glucose variability improvement 2. Improved QOL 3. Perceived as safe 4. Provider interaction experiences 5. Customizability
Quintal (2020)	Canada (33583856)	AndroidAPS, OpenAPS, Loop (Qualitative)	Interviews with 16 participants with type 1 diabetes not using DIY AID	Views on the ethical considerations raised by DIY AID; qualitative content analysis of interview transcriptions	Subcategorized; autonomy, identity, relationships, safety, privacy, public and private coverage, justice and patient selection.
Crocket (2020)	New Zealand (31646890)	AndroidAPS, OpenAPS and Loop (Qualitative)	Semi-structured interviews with 9 mentors from the DIY APS community; 4 female, 5 male, 4 people with diabetes, 5 have family members with diabetes.	Reasons for mentoring Implementation of mentoring Challenges of mentoring	Altruism Online forums Frequency of questions, dealing with conflict and managing workload.
Litchman (2020)	USA (32627587)	AndroidAPS, OpenAPS and Loop (Qualitative)	Analysis of Twitter Data 2014–2017 looking at tweets referencing OpenAPS or WeAreNotWaiting; 46,578 tweets by 7886 participants.	Conversation sentiment. Visual representations of patient-led innovation. Identification of personas who engage in DIY patient led technologies on twitter.	82–85% positive interactions Photos disseminate media and conference coverage, showcase devices, celebrate connections and accomplishment and provide instructions. Personas are; fearless leaders, loopers living it up, parents on a mission, the tech titans, movement supporters and HCP advocates.
**First Author (Year)**	**Country (PMID)**	**AID System (Study type)**	**Participants**	**Outcome Measure and Results**	
Shepard (2020)	USA (33000636)	AndroidAPS, OpenAPS, Loop (Qualitative)	Summary of a workshop with 60 stakeholders at Advanced Technologies and Treatment in Diabetes Conference Feb 2020.	User perspectives HCP perspectives Ethical considerations	No increase safety risk relative to human error. Value HCP willingness to learn about DIY AID. Limited knowledge and experience, liability and safety concerns. Off-label devices, alterations in patient-clinician relationship.
Schipp (2021)	Australia (33720767)	AndroidAPS, OpenAPS and Loop (Qualitative)	Semi-structured interview with 23 adults with T1DM using DIYAID for 1-34months, age 25–64 years, 10 female, 13 male.	Participants reported challenges with DIY AID. Participants reported support strategies.	Financial cost set-up, sourcing hardware, lack of technical knowledge, time consuming set-up, potential risks, lack of support from industry, lack of familiarity HCPs with technology, carrying multiple components, battery use, screen time. Peer support, self-sufficiency, risk management and trade-offs.
Crocket (2021)	New Zealand (34826158)	AndroidAPS (Qualitative)	Community Derived Automated Insulin Delivery study (CREATE); content analysis from fortnightly team meetings in the first 4 months of the trial. Team comprised; 5 endocrinologists, 5 diabetes specialist nurses, 2 open-source AID community members.	Key topics discussed; from review of meetings and Slack digital communication platform	AID user-interface was the most frequently reported AID specific challenge for HCP. Challenges largely related to specific devices, rather than AID. Most frequent learning challenge was insulin pump and cannula problems relating to DANA-I insulin pump (24% of conversations)
**First Author (Year)**	**Country (PMID)**	**AID System (Study type)**	**Participants**	**Outcome Measure and Results**	
Schipp (2021)	Australia (34599617)	AndroidAPS, OpenAPS and Loop (Qualitative)	Semi-structured interview; 23 adults T1D using DIY; 25–64 years, 10 F. Using DIY AID; <6 months (n = 9), 6–12 months (n = 6) or > 12 months (n = 8).	Participants key features they value in DIY AID. Benefits of these features Perspectives on future use of these systems.	Compatibility, user-led design, customizability, ability to evolve faster and community driven. Choice, solutions which meet needs, ownership, staying one-step ahead and real-time support. Collaboration with commercial products, to enable them to benefit from open-source learning.
Wong (2021)	USA (34780283)	Loop (Mixed-Methods)	46 of 874 Loop users identified as discontinuing during the observation time period. 45 completed a discontinued use survey and 19 semi-structured interviews.	Factors associated with discontinued use. Reasons for stopping. Prominent themes on qualitative analysis.	Older age and not trusting Loop. ‘I decided to try something else’ - 27.8% ‘It just didn’t help as much as I thought it would’– 22.2% Mental and emotional burden, adjusting settings, fear of disapproval, technical and logistical barriers, specific circumstances and concerns.

The majority of these studies (n = 6) reported on the outcomes with a combination of the three DIY AID system types. Participants included; users (5 studies), care givers (2 studies), HCP (2 studies), mentors in the DIY AID community (1 study, 9 participants), people with type 1 diabetes not currently using a DIY AID system (1 study, 16 participants), as well as people that had decided to stop using Loop (1 study, 45 participants).

### Conference abstracts

The 24 conference abstracts identified relating to DIY AID use, described in Table 1.5 in S1 Appendix [57–80] were published as part of ATTD (n = 10), ADA (n = 7), Diabetes UK (n = 3), Annual Diabetes Technology Meeting (n = 1), EASD (n = 1), Endocrine Abstracts (n = 1) and the International Society for Pediatric and Adolescent Diabetes (n = 1) conferences. These abstracts comprised case reports/case series (n = 5), qualitative (n = 4) and retrospective (n = 9), cross-sectional (n = 4) and prospective (n = 2) cohort studies.

#### Glycemic variability

Self-reported retrospective user data looking at glycemic variability with OpenAPS use was first published by Lewis in 2016, with 18 participants reporting improved HbA1c (mean 6.2 vs 7.1% prior to OpenAPS use) and TIR (80-180mg/dL); (81 vs 57% prior to OpenAPS) [32]. Further data on OpenAPS outcomes was published by Melmer in 2019, through retrospective analysis of 80 users’ CGM data, that had been uploaded to the OpenAPS Data Commons Repository. Of these, 34 users had additional data available from Sensor-augmented insulin pump (SAP) use prior to OpenAPS; with a mean reduction in HbA1c of 0.4% (p<0.0001) and increase in TIR of 9.3% (p<0.0001), relative to SAP use [35].

Sole use of AndroidAPS was studied in 4 of the observational studies, both retrospective (n = 2) and prospective (n = 2), including a total of 85 participants. AndroidAPS implementation ranged from a minimum of three nights, up to six months duration [34, 41, 43, 44]. The three studies with a minimum of three months AndroidAPS use, all reported improvements in HbA1c and TIR from baseline. The largest of these by Petruzelkova et al., followed 36 children; 18 pre-school (age 3–7 years) and 18 school age (age 8–14 years), for six months following switching from SAP to AndroidAPS. Glycemic outcomes improved in the pre-school children with AndroidAPS in comparison to SAP use; HbA1c 48.5 vs 53.8mmol/mol (p = 0.01) and TIR 78.6 vs 70.8% (p = 0.01). This improvement with AndroidAPS use was also demonstrated in the school age children; HbA1c 45.1 vs 52.6mmol/mol (p = 0.01) and TIR 82.9 vs 77.2% (p = 0.04) [43].

With Loop, one prospective observational study followed 558 participants for six months, after initiation of Loop. TIR (70-180mg/dL) and HbA1c were compared from baseline therapy, to after six months of Loop use with improvement seen in both of these parameters with Loop in comparison to baseline therapy; mean TIR 73 vs 67% (p<0.001) and HbA1c 6.5% vs 6.8% (p<0.001) [42].

Glycemic outcomes with a combination of DIY AID system types were reported in 6 studies, including 5 self-reported user outcome studies, and a retrospective observational cohort. Self-reported user outcomes involved a total of 1508 participants worldwide, with all studies reporting improvements in TIR and/or HbA1c with DIY AID use [5, 33, 36, 40, 47]. Jeyaventhan et al. reported retrospective observational glycemic data with six months of DIY AID use in 30 individuals (50% Loop, 36.7% AndroidAPS, 13.3% OpenAPS), comparative to the same time period for 38 users of Commercial AID (Medtronic 670G). DIY AID users demonstrated greater HbA1c reduction and improved TIR relative to Commercial AID; 0.9 vs 0.1% and 78.5% vs 68.2% [45].

The cohort studies reporting data for changes in glycemic outcomes with DIY AID use were user self-reported (n = 5) [5, 32, 35, 36] and observational (n = 6) [34, 41–45], with three of these studies stating clinician review of this glycemic data in their methodology [34, 44, 45]. Glycemic changes with DIY AID use in these studies are summarized in Tables 4 and 5. HbA1c reduction ranged in self-reported studies; from 0.4–0.9% [5, 32, 35, 36] and in observational studies; 0.3–0.9% [41–45]. Improvement in TIR, self-reported; 9.3–23% [5, 32, 35, 36, 47] and observational; 0–11.3% [34, 41–44]. In the conference abstracts, glycemic outcomes were reported in 10 observational studies, with a minimum of 1 month and maximum 11 months duration of DIY AID use. Mean HbA1c achieved ranged 6.1–6.7%, and a TIR 77.6–87.8% with DIY AID [57–59, 61–65, 68, 73]. Mean HbA1c reduction across the abstracts reporting change relative to baseline insulin delivery method; ranged 0.3–0.85%, with mean increase TIR 6.4–22.7% [57–59, 61, 62, 68]. Two abstracts compared DIY AID to CSII with Freestyle Libre use, for a minimum of one month; a mean increase of 24.8–27.7% TIR was reported with the DIY systems [63, 73].

**Table 4:** Change in HbA1c with DIY AID.

Study Type	Author (Year)	DIY AID Type	Pre-DIY HbA1c (%)	HbA1c with DIY AID (%)	Change in HbA1c with DIY AID (%)	p value (if specified)
Self-Reported	Lewis (2016)	OpenAPS	7.1	6.2	-0.9	
	Braune (2019)	All	6.91	6.27	-0.64	p<0.001
	Melmer (2019)	OpenAPS	6.6	6.2	-0.4	p<0.0001
	Braune (2021)	All	7.14	6.24	-0.9	
Observational	Wu (2020)	AndroidAPS	7.63	6.79	-0.84	p = 0.02
	Lum (2021)	Loop	6.8	6.5	-0.3	p<0.001
	Petruzelkova (2021)	AndroidAPS	53.2 mmol/mol (7.0%)	46.8 mmol/mol (6.4%)	-0.6	
	*Gawrecki (2021)	AndroidAPS	6.8	6.3	-0.5	p<0.001
	*Jeyaventhan (2021)	All	7.1	6.2	-0.9	

*Clinician review of data documented in study methodology

**Table 5:** Change in Time In Range (TIR) with DIY AID.

Study Type	Author (Year)	DIY AID Type	Pre-DIY TIR (%)	TIR with DIY AID (%)	Change in TIR with DIY AID (%)	p value (if specified)
Self-Reported	Lewis (2016)	OpenAPS	58	81	+23	
	Braune (2019)	All	64.2	80.68	+16.48	p<0.001
	Melmer (2019)	OpenAPS	71.1	80.4	+9.3	p<0.0001
	Braune (2021)	All	62.96	80.34	+17.38	
	Street (2021)	All	63.9	81.3	+17.3	
Observational	*Petruzelkova (2018)	AndroidAPS	82%	82%	0	
	Wu (2020)	AndroidAPS	75.01	84.28	+9.27	p<0.001
	Lum (2021)	Loop	67	73	+6	p<0.001
	Petruzelkova (2021)	AndroidAPS	74	80.75	+6.75	
	*Gawrecki (2021)	AndroidAPS	68	79.1	+11.3	p<0.001

*Clinician review of data documented in study methodology

#### Safety

The frequency of episodes of hypoglycemia (n = 2), severe hypoglycemia (n = 2) and TBR (n = 7) were reported in a total of 9 studies [33–35, 40–45], with improvements in these parameters reported with the use of all three DIY AID system types. TBR was reduced by 0.1–1.11% across the observational studies [41, 42, 44]. Two observational studies reported no improvement in TBR [34 and 43] using Android APS, time spent 3.0–3.8mmol/L remained static at 3% in pre-school age children, and increased from 2.6 to 3.8% in school age children using AndroidAPS for six months, relative to baseline SAP [43]. An increased TBR, with three nights AndroidAPS use in 22 children was reported relative to SAP; 5% vs 3% [34]. In user self-reported studies (n = 2), 17.9% [40] and 74% [33] of users reported a reduction in the frequency of hypoglycemia with the use of DIY AID.

Gawrecki et al., reported primary outcomes of both safety and glycemic control. No cases of Severe Hypoglycemia or Diabetic Ketoacidosis (DKA) occurred in twelve weeks of AndroidAPS use. A reduction in percentage time spent <54mg/dL (0.1%), and percentage time <70mg/dL (0.75%), was demonstrated in 12 AndroidAPS users, relative to baseline CSII [44]. Safety was assessed in 68 AID users, comparing outcomes in 38 users of Commercial and 30 DIY AID. Over six months no episodes of DKA occurred and TBR was 3.2% with DIY and 2.6% Commercial AID. This study highlighted a non-significant increase in severe hypoglycemia in users of Commercial relative to DIY AID (p = 0.104) [45].

In the 8 conference abstracts reporting TBR, mean TBR with DIY AID ranged 2.5–4.9% [57–60, 63–65, 73]. A mean reduction in TBR 0.6–6.07% relative to baseline therapy was reported [57–60, 64]. Relative to users of Freestyle Libre with CSII, DIY AID use was associated with 3.2% reduction in TBR [63, 73]. No hospital admissions or episodes of severe hypoglycemia were stated with OpenAPS use, for a mean duration of 11 months in 9 adult users [63].

#### Quality of life

Quality of life was assessed by Wu et al. in 15 participants with 3 months of AndroidAPS use, through the use of the EuroQol Five-Dimension 5-Level Health Questionnaire (EQ-5D-5L), both in the form of utility index (UI) and visual analogue scale (VAS). Improvement in mean score with AndroidAPS use, was seen in VAS relative to baseline; 82 vs 77 (of a maximum 100), p = 0.13, but no change demonstrated in UI, mean 0.88 vs 0.88 (of a maximum 1). With UI calculated on a 0 to 1 scale (no to severe impairment/unable) scored through questions in the five dimensions of mobility, self-care, usual activities, pain/discomfort and anxiety/depression. A small improvement was seen in diabetes-related distress; an increase from 6 to 9% (p = 0.143) in those scoring little or no diabetes distress, and a reduction in fear of hypoglycemia relative to baseline therapy through use of the Hypoglycemia Fear Survey II- Worry Scale (HFS-II); mean score 22.13 vs 26.27 (lower score better, of a maximum 72), p = 0.01 [41]. Self-reported improved sleep quality was highlighted by 94% of OpenAPS users [32], and 79% of DIY AID users [33]. When questioned regarding the main benefits of DIY AID, 22.5% of 86 participants, reported better sleep quality/nightly safety [40]. Assessment of the motivations to commence DIY AID, revealed 72% of adults and 80% of caregivers cited improved sleep quality as a motivating factor for this choice of insulin delivery system [5].

An abstract for ADA by Hood et al., assessed quality of life outcome measures both before and after 3 months of using Loop, in 254 new Loop users. Improvements were seen in mean scores of diabetes-related distress, as measured by the diabetes distress scale (DDS); 2.06 to 1.66 (scored from 1; not a problem, to 6; a very significant problem). A reduction in fear of hypoglycemia (HFS-II); 19.74 to 17.18, and improvement in sleep were also demonstrated, (Pittsburgh Sleep Quality Inventory, scored 0 to 21); 6.82 to 5.39 [66]. In a cross-sectional study abstract for ATTD, Zabinsky et al. explored self-reported outcomes with DIY AID in 180 users; 74.7% reported improved sleep quality/quantity, 69.4% reduced time spent managing diabetes and 76.9% reduced diabetes-related stress [67].

#### Healthcare provider (HCP) attitudes

HCP opinions on DIY AID use were collected in 4 studies, 3 from the USA and one from the UK, with a total of 753 HCP surveyed [37–39, 46]. In the UK study of 317 HCPs, 91% of participants advised they would not initiate discussions about DIY AID and 2% reported that they perceived the systems as dangerous [38]. One study from the USA, surveyed 152 HCP approached via the American Association of Diabetes Educators, 27% reported that they perceived these systems as safe [39]. A lack of understanding in how the systems work was reported by 74.4% of participants, 11.6–34.9% felt comfortable answering questions about DIY AID systems [37] and 97% reported a willingness to learn more about them [38]. Fear of HCP disapproval of DIY AID was reported as a prominent reason for users who had decided to stop using Loop [56]. In a survey of 104 school nurses, 23% reported a child using DIY AID attended their school, 46% stated they had no prior knowledge of DIY AID and 96% felt the child should be able to share their data with a parent or guardian during the school day [46]. HCP supporting AndroidAPS use as part of the Community Derived Automated Insulin Delivery study (CREATE) in New Zealand [81, 82], found that user challenges with this system most frequently related to device issues (the insulin pump and cannula in 24% of analyzed conversations), as opposed to DIY AID specific challenges [54].

Cohen et al. reported a qualitative interview study of the perceived benefits and barriers of DIY AID use in 20 HCP working in pediatric and adult diabetes services in the UK, as an abstract for ATTD. Of the 20 participants, 19 reported liability concerns and lack of formal guidelines to be barriers to supporting the current use of DIY AID in widespread clinical practice [76].

#### Social media

Attitudes from users and the DIY AID community were collected through 6 studies using social media (Facebook and Twitter), with 2 reporting the content of Tweets [48, 51], and 4 using these platforms to distribute surveys to DIY AID users [33, 36, 39, 47]. A total of 49,925 tweets were analyzed from 8214 participants [48, 51]. User opinions of DIY AID across these studies were positive, with 82–85% positive interactions on Twitter [48]. Through an interview study, mentors in the DIY AID community, largely through the use of social media platforms, reported altruism as the main reason behind their role, and the frequency of questions and managing workload, to be the biggest challenges they face [50].

Of the conference abstracts reviewed, 2 detailed the recruitment of participants through the use of social media. A qualitative interview study of 11 girls and women, discussing glycemic variability and need for algorithm adjustment relating to hormonal changes, recruited study participants through topic related discussion on social media [75]. Girelli et al., distributed a survey via the Looped and OpenAPS Facebook groups, gaining responses from 120 respondents interested in DIY AID use and 19 current users [70].

## Discussion

The findings of this scoping review highlight that there is a large, and rapidly expanding body of published outcome data relating to DIY AID system use. We have reviewed an extensive scope of outcome studies, varying in aims and methodology. With the nature of research into these rapidly evolving systems, there is no doubt that at the date of publication of this review, new insights into the field will already be available. This may be particularly apparent with the lack of DIY system subtype specific terminology in our search terms (Android or OpenAPS), and especially in the use of newer DIY system types (including FreeAPS and AI APS). However, through this review we have highlighted evidence of impressive and consistent glycemic outcomes with all forms of DIY AID use; improvements in TIR, HbA1c and TBR, and have seen no great discrepancies between the outcomes reported in observational studies relative to self-reported data [5, 32, 35, 36, 41–45, 47].

Social media platforms have provided both a source to gather large quantities of user data, as well as a means for recruitment of study participants. In addition, we have found online resources and social media, especially Facebook and Twitter, to be a large part of the support structure for people using DIY AID [50, 53], with the Looped Facebook group now having over 28,000 members [83]. Supporting safety and efficacy data are additionally published by users themselves through internet resources, notably in the form of blogs [84]. This online community however, is not a conventional method to collect and publish data relating to pharmaceutical and technological advances, and will bring the validity of the reported results into question, with the prospect of selection bias in addition to the seemingly very favorable user outcomes being self-reported.

Current users of DIY AID systems are in themselves self-selected, the systems are user-built and individualized. The development of the technology in DIY AID has been driven by the user from its outset, rather than a pharmaceutical company, making it challenging to collect impartial and externally valid data on its use, as well as to fund large clinical trials [3]. This individualized user choice in method of insulin delivery for management of type 1 diabetes, brings into question both the appropriateness and the utility of randomized control trials (RCT), in the setting of these systems.

It is challenging to compare results between the studies reviewed, as well as between the DIY AID system types, due to the variable design and duration of the studies described, in addition to the inclusion criteria for participants. We have reviewed observational data relating to the use of AndroidAPS (85 participants) [34, 41, 43, 44], with three of these studies implementing the system for a minimum of three months (63 participants), all demonstrating improvement in mean HbA1c and TIR [41, 43, 44]. An observational study of Loop (558 participants) from the USA highlighted similar improvements in HbA1c (0.3%), TIR (7%) and a reduction in TBR (0.1%). Notably these observational improvements in glycemic outcomes, were all within in the first six months of Loop use, in individuals with already close to optimal glycemic control; mean HbA1c at baseline 6.8% [42]. These glycemic outcomes are not representative of the ‘average’ person with type 1 diabetes, with just 21% of American adults with type 1 diabetes reaching an HbA1c of less than 7% (53mmol/mol) [85]. This reiterates the concept that users of DIY AID do not represent a ‘typical’ person with diabetes, and that the outcomes demonstrated with these systems in observational studies are largely a reflection of the individual that is choosing to use this technology, in conjunction with the benefits of the system itself. There is questionable generalizability of the results across the studies we have reviewed, to the implementation of these systems in the wider population of people with diabetes. Despite the overwhelmingly positive outcomes in both the observational studies and self-reported data, we cannot infer from this that these systems are the optimal glucose management system for all people with type 1 diabetes.

In order to set up these seemingly very beneficial systems, a level of understanding of both the technology and type 1 diabetes management is needed. DIY AID does not remove the requirement for user input in the management of type 1 diabetes; understanding of carbohydrate counting, insulin:carbohydrate ratios and insulin sensitivity factors, the technology alone is not enough [86]. In addition to diabetes education, a level of both literacy and numeracy skills are needed to follow the instructions for system set up and to overcome any barriers that may be faced in this process [87–89]. These resources guide users in customisation of settings, with variable DIY AID algorithm types available to meet user needs [1]. The studies reviewed highlight the importance of internet resources, social media platforms as well as mentors in the DIY AID community in guiding users through the set up and any on-going challenges with these systems. This community of users and advisors have much greater experience in the use of these systems than the majority of HCP [8, 83].

Currently, the appropriate role of HCP in DIY AID is not clear and the studies of HCP knowledge and attitudes in the use of these systems reflect this uncertainty. Due to the novelty and rapidly evolving nature of these systems, the majority of HCP caring for people with diabetes have not received any formal system-specific education whilst training for their roles. With the rising popularity of DIY AID worldwide, the majority of HCP working in the specialty of Endocrinology and Diabetes, are now likely to be the responsible clinician for one or more person using some form of DIY AID [8]. With a lack of training in their use, as well as unresolved ethical and potentially medico-legal concerns, it is unsurprising that the majority of HCP are not voluntarily broaching the subject of DIY AID systems in consultations with people with type 1 diabetes [38]. A lack of system-specific training, and open communication with potential future as well as current DIY AID users, results in compromised and inconsistent care for people with diabetes. The OPEN consensus statement was not available at the time the studies we have reviewed took place [14]. Further HCP opinion studies going forward may reflect greater confidence in discussing this potential management option with people with diabetes and/or their caregivers, in line with this guidance. However, there are increasing capabilities and available options in Commercial forms of AID, if these ongoing advancements meet user needs there may be a reduction in future DIY AID use, with HCP potentially more likely to actively support users choosing a regulatory approved glucose management system.

Active support of an ‘off-label’ medical device, such as DIY AID, requires strong clinical evidence which is, defined as a minimum of one RCT in Canada [90]. Despite the broad scope of evidence to support DIY AID use highlighted by this review; beneficial glycemic outcomes, quality of life measures and supportive safety data, no RCT has been completed. An RCT protocol has been completed in New Zealand; comparing six months of Android APS to Sensor Augmented Pump therapy, with results currently awaited [81]. This study was funded by the Health Research Council of New Zealand and includes both children and adults [82]. The OPEN consensus statement makes reference to the difficulties we have discussed, in performing an RCT in the use of DIY AID, suggesting the extensive real-world data available, that we have highlighted in this scoping review, should be considered in regulatory approval processes [14].

## Conclusion

There is a vast and rapidly expanding body of observational and self-reported user data available in the use of DIY AID systems. There are however substantial potential weaknesses in these studies, with inclusion of biased samples presenting challenges in the generalizability of this real-world evidence. No RCT data is currently available for any of the DIY AID system types; the standardized method to achieve conclusive, unbiased, level 1 evidence. This may however not be the optimal data collection methodology in the assessment of outcomes with this self-selecting, individualized and user-built technology choice. In contrast to the evidence supporting commercially available AID systems, an objective unbiased data deficiency for DIY AID and lack of regulatory approval is resulting in uncertainty worldwide among HCP. Education and best practice recommendations for HCP are lacking, in the utilization of DIY AID systems. These interventions are imperative to enable appropriate and optimal HCP support for people with type 1 diabetes choosing to use these glucose management systems. Mentors within the DIY AID community have been highlighted as rich knowledge sources, who could play a key and essential role, in developing focused education and training programs, in this exciting and rapidly expanding field.

## Supporting information

S1 Appendix (DOCX)

S1 Checklist Preferred Reporting Items for Systematic reviews and Meta-Analyses extension for Scoping Reviews (PRISMA-ScR) checklist. (DOCX)
