# Supplementary material for: A scoping review of Do-It-Yourself Automated Insulin Delivery system (DIY AID) use in people with type 1 diabetes
Source: PLoS One. 2022 Aug 11;17(8):e0271096. doi: 10.1371/journal.pone.0271096 (PMC9371307; doi:10.1371/journal.pone.0271096)
Supplement: S1 Appendix — (DOCX) [file pone.0271096.s001.docx]

# **Appendices**

**Table 1.1 Study Quality Assessment – Critical Appraisal Skills Program Checklist Qualitative studies** [48-56]

| **Author**  **(Year)** | **Clear statement of aims** | **Appropriate methodology** | **Appropriate research design** | **Appropriate recruitment strategy** | **Appropriate data collection methodology** | **Considered relationship between researcher and participant** | **Considered ethical issues** | **Rigorous data analysis** | **Clear findings** |
| --- | --- | --- | --- | --- | --- | --- | --- | --- | --- |
| Litchman  (2019) | Yes | Yes | Yes | Yes | Yes | Can’t tell | Yes | Yes | Yes |
| Quintal  (2020) | Yes | Yes | Yes | No | Yes | Can’t tell | Yes | Yes | Yes |
| Crocket  (2020) | Yes | Yes | Yes | Yes | Yes | Yes | No | Yes | Yes |
| Litchman  (2020) | Yes | Yes | Yes | Yes | Yes | Can’t tell | Yes | Yes | Yes |
| Shepard  (2020) | Ye | Yes | Yes | Yes | Can’t tell | Can’t tell | No | No | Yes |
| Schipp  (2021) | Yes | Yes | Yes | Yes | Yes | Can’t tell | Yes | Yes | Yes |
| Crocket  (2021) | Yes | Yes | Yes | Yes | Yes | Can’t tell | Yes | Yes | Yes |
| Schipp  (2021) | Yes | Yes | Yes | Yes | Yes | Can’t tell | Yes | Yes | Yes |
| Wong  (2021) | Yes | Yes | Yes | Yes | Yes | Can’t tell | Yes | Yes | Yes |

**Table 1.2 Study Quality Assessment – Critical Appraisal Skills Program Checklist Cohort studies** [32, 34-36, 41-45]

| **Author**  **(Year)** | **Clear statement of aims** | **Appropriate recruitment** | **Accurate measurement of exposure** | **Accurate measurement of outcome** | **Confounders:**  **a) Identification**  **b) Consideration in analysis** | **Follow-up:**  **a) complete**  **b) duration** | **Appropriate results** | **Results applicable to local population** | **Results consistent with existing evidence** | **Clear implications for practice** |
| --- | --- | --- | --- | --- | --- | --- | --- | --- | --- | --- |
| Lewis  (2016) | Yes | Yes | Yes | Can’t tell | 1. no 2. no | a) can’t tell  b) can’t tell | Yes | Can’t tell | Can’t tell | Can’t tell |
| Petruzelkova  (2018) | Yes | Yes | Yes | Yes | 1. yes 2. no | a) yes  b) can’t tell | Yes | Yes | Yes | Yes |
| Melmer  (2019) | Yes | Yes | Yes | Yes | 1. no 2. no | a) can’t tell  b) can’t tell | Yes | Can’t tell | Yes | Can’t tell |
| Braune  (2019) | Yes | Yes | Yes | Yes | 1. yes 2. no | a) yes  b) can’t tell | Yes | Yes | Yes | Can’t tell |
| Wu  (2020) | Yes | Yes | Yes | Yes | 1. yes 2. no | a) yes  b) yes | Yes | Yes | Yes | Yes |
| Lum  (2021) | Yes | Yes | Yes | Yes | 1. yes 2. no | a) yes  b) yes | Yes | Yes | Yes | Yes |
| Petruzelkova (2021) | Yes | Yes | Yes | Yes | 1. yes 2. no | a) yes  b) yes | Yes | Yes | Yes | Yes |
| Gawrecki  (2021) | Yes | Yes | Yes | Yes | 1. yes 2. no | a) yes  b) yes | Yes | Yes | Yes | Yes |
| Jeyaventhan  (2021) | Yes | Yes | Yes | Yes | 1. no   b) no | a) yes  b) yes | Yes | Yes | Yes | Yes |

**Table 1.3 Study quality Assessment – Appraisal Tool for Cross-sectional Studies (AXIS)** [5,33,37-40,46,47]

| **Author**  **(Year)** | **Clear aims** | **Study design, sample, target pop.** | **Risk factor and outcome variables** | **Statistical methods described** | **Description of data** | **Response bias considered** | **Justified**  **Discussion** | **Other incl. ethical approval** |
| --- | --- | --- | --- | --- | --- | --- | --- | --- |
| Hng  (2018) | Yes | Yes | Yes | No | Yes | Yes | Yes | Don’t know |
| Murray  (2020) | Yes | Yes | Yes | Yes | Yes | Don’t know | Yes | Yes |
| Crabtree  (2020) | Yes | Yes | Yes | No | Yes | Don’t know | Yes | Don’t know |
| Palmer  (2020) | Yes | Yes | Yes | Yes | Yes | Yes | Yes | Don’t know |
| Herzog  (2020) | Yes | Yes | Yes | Yes | Yes | Don’t know | Yes | Yes |
| March  (2021) | Yes | Yes | Yes | No | Yes | Yes | Yes | Yes |
| Braune  (2021) | Yes | Yes | Yes | Yes | Yes | Yes | Yes | Yes |
| Street  (2021) | Yes | Yes | Yes | Yes | Yes | Yes | Yes | No |

**Table 1.4 Enhancing Transparency in Reporting the Synthesis of Qualitative Research (ENTREQ) Statement**

| **No.** | **Item** | **Description** |
| --- | --- | --- |
| 1 | Aim | A review of the currently available literature surrounding DIY AID systems, specifically to highlight the current evidence to support their use. |
| 2 | Synthesis methodology | Thematic synthesis due to variety of study methodologies with similar outcomes assessed |
| 3 | Approach to searching | Comprehensive search strategy (fig 2) to identify all relevant publications published studies and conference abstracts |
| 4 | Inclusion criteria | Studies published in English, relating to the use of DIY AID systems, qualitative, cross-sectional and cohort |
| 5 | Data sources | Embase, Medline, Web of Science, Scopus, Cochrane library, Proquest, conference abstracts ADA, DUK, ATTD last 2 years. |
| 6 | Electronic search strategy | Figure 2 - search strategy performed on 31^st^ December 2021 |
| 7 | Study screening methods | Two independent reviewers (AM and KC), screening by title, abstract and full text (figure 1- PRISMA flowchart) with independent third reviewer (AL) to resolve any debated studies for inclusion. |
| 8 | Study characteristics | Table 1 – study characteristics case control studies  Table 2 - study characteristics cohort studies  Table 3- study characteristics qualitative studies  Table 1.5 (appendices)- study characteristics conference abstracts |
| 9 | Study selection results | See figure 1 PRISMA Flowchart |
| 10 | Rationale for appraisal | Study quality assessment; critical appraisal skills program checklist for both qualitative and quantitative studies (CASP), appraisal tool for cross-sectional studies (AXIS). |
| 11 | Appraisal items | Study design, data analysis and reporting presented as study characteristics in table 1, 2, 3, and table 1.5 appendices. |
| 12 | Appraisal process | Appraisal process performed by one independent author (AM) |
| 13 | Appraisal results | Study quality assessment; Appendices table 1.1, table 1.2 and table 1.3. |
| 14 | Data extraction | Outcomes and results were manually extracted from the studies after reading the full text and summarized in table 1, table 2, table 3 and table 1.5 (appendices). |
| 15 | Software | N/A |
| 16 | No. of reviewers | Analysis of studies performed by two independent reviewers (AM and KC), with third reviewer (AL) for any disputes in inclusion. |
| 17 | Coding | Study results were analyzed line by line to search for common themes or concepts |
| 18 | Study comparison | Studies were compared in participants, methods, outcomes and results |
| 19 | Derivation of themes | Deriving the themes was an inductive method, using the study outcomes to derive discussion themes due to the limited quantity of existing research in this field |
| 20 | Quotations | DIY AID system users, community members and HCPs provided quotations in the form of open-response survey questions and semi-structured interviews. |
| 21 | Synthesis output | Discussions and conclusions of this manuscript |

**Table 1.5 DIY AID Conference Abstracts** [57-80]

| **First Author (Year)** | **Country**  **(Format)** | **System**  **(Study type)** | **Participants** | **Outcome Measure and Results** | |
| --- | --- | --- | --- | --- | --- |
| Lewis  (2018) | USA  (ADA abstract) | OpenAPS  (Retrospective cross-over) | 20 OpenAPS users, 4-6 weeks before and after OpenAPS. Mean age 30.2 yr, mean diabetes duration 18.4 yr | With OpenAPS use;  Mean blood glucose  TIR  TAR  TBR  HbA1c | vs pre-OpenAPS  128.3 vs 135.7 mg/dL  82.2 vs 75.8%  13.3 vs 18.3%  4.5 vs 6.0%  6.1 vs 6.4% |
| Choi  (2018) | Korea  (Annual Diabetes Technology Meeting abstract) | OpenAPS  (Retrospective) | 10 OpenAPS users; median age 9.5yrs, 5 male, median duration OpenAPS 30 days, | With OpenAPS use;  HbA1c  TIR  TAR  TBR | vs pre-OpenAPS  6.2 vs 6.8%  82.8 vs 65.1%  12.3 vs 24.5%  4.9 vs 5.4% |
| Choi  (2018) | Korea  (ADA abstract) | OpenAPS  (Retrospective) | 20 people with T1DM (50% male), mean age 11.9 yrs. Mean duration OpenAPS 120 days (30-240) | With OpenAPS use;  HbA1c  TIR  TAR  TBR | vs pre-OpenAPS  6.3 vs 6.8%  83.3 vs 70.1%  13.3 vs 24.7%  3.4 vs 5.1% |
| Provenzano  (2018) | Italy  (ADA abstract) | OpenAPS  (Retrospective) | 30 people with T1DM (19 male, 11 female), mean age 35.9 years. Self-report data pre and post 3 months OpenAPS | With OpenAPS use;  HbA1c  TBR  Safety | vs pre-OpenAPS  6.61 vs 7.17%  2.48 vs 8.55%  No serious AE reported |
| Braune  (2019) | Germany  (ADA abstract) | OpenAPS, AndroidAPS, Loop  (Cross-sectional) | Online survey, 1058 participants; 80.2% adult users with median age 41 years, 19.8% caregivers for children with T1DM. | With DIY AID use;  HbA1c  TIR  Cost of DIY (out of pocket)/yr  Motivations for using a DIY AID system. | vs pre-DIY  6.24 vs 7.07%  83.07 vs 63.21%  712 USD  Improved glycemic control, need for ‘auto-pilot’, less complications, better sleep for caregivers. |
| **First Author (Year)** | **Country**  **(Format)** | **System**  **(Study type)** | **Participants** | **Outcome Measure and Results** | |
| Koutsovasilis  (2019) | Greece  (EASD) | OpenAPS  (Prospective) | 51 people with T1D; 28 continued CSII and 23 commenced OpenAPS for 6 months | With OpenAPS use;  HbA1c baseline (%)  HbA1c 3 months (%)  HbA1c 6 months (%)  Total daily insulin(units)  Total bolus insulin (units)  Total basal insulin (units) | vs continued CSII users  7.85 vs 7.81  7.06 vs 7.48  6.70 vs 7.37  48.28 vs 57.74 (p=0.036)  23.03 vs 29.87 (p= 0.040)  25.54 vs 33.08 (p= 0.031) |
| Wilmot  (2019) | UK  (ADA abstract) | OpenAPS  (Retrospective) | Comparison 9 users OpenAPS; mean age 44.2, diabetes duration 25yrs, OpenAPS 11months, with 30 Freestyle Libre+CSII users, mean age 59.3, diabetes duration 26yrs, 5.9 months FSL+CSII. | With OpenAPS use;  HbA1c pre OpenAPS use  HbA1c  TIR  TBR  Safety | vs CSII and Freestyle Libre users  7.3 vs 7.6%  6.2 vs 7.2%  83.6 vs 55.9%  2.5 vs 5.7%  No hospital admission/SH either grp |
| Jiranova  (2019) | Czech Republic  (ATTD abstract) | AndroidAPS  (Retrospective cohort) | 22 children; age 3-14 years, mean duration AndroidAPS 8.7months, at least 3 months of AndroidAPS was compared to the preceding 3 months of SAP in these participants. | With AndroidAPS use;  HbA1c  TIR  TBR | vs SAP  47 vs 52 mmol/mol  83.6 vs 67.6%  4.4 vs 5.2% |
| Zabinsky  (2020) | USA  (ADA abstract) | OpenAPS, AndroidAPS, Loop  (Retrospective) | DIY group; 74 individuals, 90 days of data, mean age 36 and diabetes duration 24 years, with 98 age matched SAP participants. | With DIY AID use;  TBR  hypoglycemic episodes per month,  <54mg/dL  Mean glucose  TAR  TIR | vs SAP  3.8 vs 4.7%  32.9 vs 33.4 episodes  0.8 vs 1.3%  134.9 vs 150.3 mg/dL  16.9 vs 26.1%  79.3 vs 69.2% |
| **First Author (Year)** | **Country**  **(Format)** | **System**  **(Study type)** | **Participants** | **Outcome Measure and Results** | |
| Hood  (2020) | USA  (ADA abstract) | Loop  (Prospective) | 254 new loop users recruiting through online posting and Loop RileyLink packaging, mean age 38.4 years, HbA1c 6.64%, TIR 69.6%. | PRO surveys at 3 months;  Diabetes Distress Scale  Technology attitudes  Fear of hypoglycemia  Hypoglycemia confidence  Technology problem solving  Pittsburgh sleep quality inventory. | vs baseline  1.66 vs 2.06  20.21 vs 20.11  17.18 vs 19.74  29.91 vs 27.21  29.36 vs 28.7  5.39 vs 6.82 |
| Zabinsky  (2020) | USA  (ATTD abstract) | OpenAPS, AndroidAPS, Loop  (Cross-sectional) | 180 DIY AID users, mean age 34 yrs, duration DM 20 yrs. | Self-reported outcomes with DIY AID; Reduction in hypoglycemia Reduction in hyperglycemia  Increased sleep quality/quantity  Reduced time spent managing diabetes  Reduced diabetes related stress  Satisfied with o/n BG  Dissatisfaction with set up Troubleshooting | 86.5%  87.6%  74.7%  69.4%  76.9%  97.6%  19.9%  19.3% |
| Wu  (2020) | China  (ATTD abstract) | AndroidAPS  (Retrospective) | 10 participants with >3months AndroidAPS use; 6 female, median age 34.1yrs, diabetes duration 13 yrs, HbA1c 7.3% | With 3months AndroidAPS use;  HbA1c  TIR | vs pre-DIY  6.53 vs 7.37%  84.75 vs 76.30%  Less hypoglycemia and lower fear of hypoglycemia with AndroidAPS use. |
| Garfinkel  (2020) | Canada  (ATTD abstract/  PhD thesis) | Loop  (Case report) | Case study of an individual living in 2019 with T1DM on Loop. | Diary/literary memoir of experiences. | Opening possibility of broader empathy, the ‘second person perspective’ |
| **First Author (Year)** | **Country**  **(Format)** | **System**  **(Study type)** | **Participants** | **Outcome Measure and Results** | |
| Girelli  (2020) | Italy  (ATTD abstract) | OpenAPS (2 users), AndroidAPS (n=8), Loop (n=9)  (Cross-sectional) | Online survey via the Looped and OpenAPS Facebook groups. Respondents; 120 interested in DIY AID and 19 users, mean age 28.1 yrs. | Type of pump used.  Reasons for planning DIY AID.  Clinic response to plans. | Omnipod (50%), AccuChek (25%), Medtronic (15%), Tandem (5%), Dana (5%).  Improve control, sleep, more discrete, reduce hypoglycemia.  60% positive |
| White  (2020) | UK  (Diabetes UK abstract) | Loop  (Case report) | 37yr F with 22yr history of T1DM, started looping eight months prior to 2^nd^ pregnancy (1^st^ pregnancy CSII and CGM) | With Loop use;  Preconception HbA1c  Final pregnancy HbA1c  Delivery type and timing  Birth weight | vs CSII and CGM  6.2 vs 6%  5.3 vs 5.5%  C-section 38+4 vs normal 38+4 wk  3.53 vs 3.79kg |
| Volkova  (2020) | Russia  (Endocrine Abstracts) | OpenAPS, AndroidAPS, Loop  (Cross-sectional) | 88 people with T1DM or their caregivers, using Loop (19.3%), AndroidAPS (50%) and OpenAPS (30.7%) | Reported advantages DIY AID;  Increase TIR  Decrease mild hypoglycemia Decrease severe hypoglycemia  Improved nightime glycemia | 78%  61%  59%  86% |
| Patel  (2021) | UK  (Diabetes UK abstract) | OpenAPS, AndroidAPS, Loop  (Retrospective) | 17 patients using DIY AID and 149 using CSII with Freestyle Libre, with minimum 1 month therapy | With DIY AID use;  HbA1c  TIR  TBR  TAR | vs CSII and Freestyle Libre users  47.2 vs 60.1 mmol/mol  77.6 vs 52.8%  2.5 vs 5.7%  18.8 vs 41.7% |
| Patel  (2021) | UK  (Diabetes UK abstract) | OpenAPS, AndroidAPS, Loop  (Qualitative) | 17 patients using DIY AID, mean age 43 yrs. | Free-text feedback on the use of the technology and review of patient records for patient opinions or comments | Themes identified; general quality of life (QOL), diabetes related QOL, technological problems, perception of improved diabetes control |
| Mewes  (2021) | Germany  (Abstract IS Paediatric and Adolescent Diabetes) | OpenAPS, AndroidAPS, Loop  (Qualitative) | 11 girls and women recruited through topic related discussion groups on social media; 1 during pregnancy, 1 puberty and 3 menopause. | Semi-structured interviews, focused on perceived changes, therapy adjustments, and suggestions for AID optimization. | All noted glycemic variability with menstrual cycle, concerns over algorithm adjustment due to individual nature of hormone activity. |
| **First Author (Year)** | **Country**  **(Format)** | **System**  **(Study type)** | **Participants** | **Outcome Measure and Results** | |
| Cohen  (2021) | UK  (ATTD abstract) | OpenAPS, AndroidAPS, Loop  (Qualitative) | 20 HCPs from pediatric and adult diabetes services, interviewed on perceptions of DIY AID benefits and barriers to its use. | Reported benefits;  Improved glycemic outcomes  No added risk compared to other diabetes tech  Customizability  Barriers; Liability concerns  Lack of formal guidelines | n=13  n=12  n=9  n=19  n=19 |
| Dowden  (2021) | UK  (ATTD abstract) | AndroidAPS  (Case report) | 37 yr female completing 1200km PBP cycle using AndroidAPS. | Cycle completed  TIR, TAR, TBR  <3.5mmol/L, lowest glucose  Mean glucose | 89 hrs 28mins  58%, 38%, 4%  <1%, 3.1mmol/L  9.7mmol/L |
| Cohen  (2021) | UK  (ATTD abstract) | OpenAPS and AndroidAPS  (Qualitative) | Semi-structured interviews with 26 adults and 14 parents of youth (<18yrs) using DIY AID. | Benefits for adults  Benefits for parents  Shortcomings for adults  Shortcoming for parents  HCP support | Glycemic (n=26), overnight management (n=26), reduced burden diabetes (n=23)  Overnight (n=12), glycemic (n=11), exercise (n=10)  Independent set up (n=23), system only as good as components (n=18), insurance (n=18)  Set up (n=10), components (n=10), connectivity (n=6)  Majority supportive |
| Alidibbiat  (2021) | Kuwait  (ATTD abstract) | Loop (n=3) and AndroidAPS (n=2) and CamAPS (n=1)  (Case series) | 5 DIY and one commercial AID user, mean age 33.7yrs, diabetes duration 23.5 yrs, BMI 23.6, HbA1c 6.3%. | DIY AID use during Ramadan;  Mean glucose  Coefficient of Variability  TIR  TBR  Days fasted  Days fast broken due to diabetes | 7.0mmol/L  28.5%  88.8%  2.5%  27.3 days  1 day |
| Treiber  (2021) | Austria  (ATTD abstract) | Loop  (Case report) | 32-year female with 16-year history of diabetes, using Loop during pregnancy, complicated by hyperemesis. | With Loop use;  HbA1c TM 1/2/3  TIR (70-140mg/dL)  TDD insulin TM 1/2/3  Delivery type  Birth weight, Timing,  APGAR. | 32/24/31mmol/mol  75-82%  28/41/61 IU  C-section  3590g, 40+3 weeks  9/10/10.  Noted CGM inaccuracy with HG and dehydration. |
